# Epidural Sustained Release Ropivacaine Prolongs Anti-Allodynia and Anti-Hyperalgesia in Developing and Established Neuropathic Pain

**DOI:** 10.1371/journal.pone.0117321

**Published:** 2015-01-24

**Authors:** Teng-Fei Li, Hui Fan, Yong-Xiang Wang

**Affiliations:** King’s Lab, Shanghai Jiao Tong University School of Pharmacy, Shanghai, China; University of Texas Medical Branch, UNITED STATES

## Abstract

Ropivacaine is a local anesthetic widely used for regional anesthesia and epidural analgesia, but its relatively short duration limits its clinical use. A novel sustained release lipid formulation of ropivacaine has been recently developed to prolong its duration. We examined the epidural anti-hypersensitivity and preemptive effects of ropivacaine in mesylate injection and sustained release suspension forms in a rat model of neuropathy produced by peripheral nerve injury. Epidural administration of ropivacaine mesylate injection specifically blocked mechanical allodynia and thermal hyperalgesia by approximately 50% with a biological half-effective duration of approximately 3 hrs. The equivalent dose of ropivacaine free-base in sustained release suspension significantly prolonged the duration of anti-allodynia and anti-hyperalgesia by approximately 2 times. Multiple daily epidural injections of ropivacaine in both the mesylate injection and sustained-release suspension forms did not induce tolerance or potentiation to anti-allodynia or anti-hyperalgesia. Moreover, the single or multiple daily administration of ropivacaine mesylate injection before surgery in particular, markedly blocked the initiation and development of neuropathic pain, increasing the biological half-effective duration from less than 4 hrs up to 1 or 2 days. The single and multiple daily epidural injection of ropivacaine sustained release suspension further delayed the biological half-lives to 2 and 3 days, respectively. Our results indicate that the epidural administration of ropivacaine effectively blocks neuropathic pain without the induction of analgesic tolerance, and significantly delays the development of neuropathy produced by peripheral nerve injury. Epidural ropivacaine sustained release suspension produces much longer blockade effects of mechanical allodynia and heat hyperalgesia, and more significantly delays the development of neuropathic pain.

## Introduction

Local anesthetics, administered via the peripheral or central route, are widely used for regional anesthesia and control of postoperative pain and chronic pain, including neuropathic pain [1]. Ropivacaine, which is chemically homologous to bupivacaine and mepivacaine, belongs to the class of long-acting amide-type local anesthetics [2]. It shows better sensory-motor differential blockade, less central nervous system and cardiovascular toxicities or side-effects, and longer duration than bupivacaine and other agents in the class [3–6]. Epidural ropivacaine at concentrations of 0.2–1% has been increasingly used in clinical practice to provide regional anesthesia and postoperative pain relief [5, 6]. However, the duration of epidural ropivacaine at the injection site is relatively short, and high and rapid systemic absorption results in low intrathecal bioavailability (around 10%) with short duration (t_1/2β_: approximately 100 min) [7, 8]. Wide clinical use of ropivacaine has prompted the development of appropriate delivery systems and different administration regimens that circumvent the problem of rapid clearance from the injection site into systemic circulation [9].

Slow release formulations using lipid vehicles, such as iophendylate, ethyl oleate, sesame oil, castor oil and bean oil, prolong spinal anesthesia and analgesia, probably through the slow but sustained release of the anesthetic from the lipid depot [10–12]. A novel sustained release system using castor oil suspension was recently been developed to delay systemic absorption. When ropivacaine free-base was dissolved in benzyl alcohol, and subsequently suspended in the injectable castor oil, a longer duration effect was obtained due to its slow but sustained release from the oil phase. Pharmacokinetic studies have shown that the elimination half-life of ropivacaine from sustained release suspension is significantly longer than that from mesylate injection in animals [11].

We examined the antinociceptive effects of the epidural injection of ropivacaine sustained release suspension compared to those of ropivacaine mesylate injection in this study. A rat model of spinal nerve injury-induced neuropathic pain was selected because it develops robust spontaneous pain, allodynia and hyperalgesia after surgery and represents human-like neuropathic pain [13]. We first tested the motor blockade by epidural injection of ropivacaine in the mesylate injection and sustained release suspension forms to determine appropriate time points to measure pain thresholds. We then examined the anti-allodynic and anti-hyperalgesic effects of single and multiple daily epidural injection of both forms in established neuropathic pain. Finally, we examined the preemptive effects of single and multiple daily effects of both ropivacaine forms on initial and developing neuropathic pain. Our results indicate that epidural administration of ropivacaine effectively blocks neuropathic pain (both mechanical allodynia and heat hyperalgesia) without induction of analgesic tolerance and significantly delays the development of neuropathic pain produced by peripheral nerve injury. More importantly, epidural ropivacaine sustained release suspension produces much longer anti-allodynia and anti-hyperalgesia, and a preemptive effect on the development of neuropathic pain.

## Materials and Methods

### Drugs

Ropivacaine mesylate injection and ropivacaine sustained release suspension were manufactured by Xi’an Libang Pharmaceutical Co. (Xi’an, China). Ropivacaine mesylate injection was freshly diluted with sterile normal saline solution (Sinopharm Group Chemical Reagent Co., Shanghai, China), and ropivacaine sustained release suspension was diluted with a sterile oil vehicle containing benzyl alcohol, benzyl benzoate and castor oil (Xi’an Libang Pharmaceutical Co.).

### Experimental animals

Adult male Wistar rats (240 ± 20 g body weight) were purchased from the Shanghai Experimental Animal Institute for Biological Sciences (Shanghai, China). Animals were housed four per cage with thick sawdust bedding at standard room temperature, under a 12/12 hrs reversed light—dark cycle (7:00 a.m.-7:00 p.m.) at a constant temperature of 22 ± 2°C. All rats received food and water *ad libitum*. The research protocols were approved by the Animal Care and Welfare Committee of the Shanghai Jiao Tong University School of Pharmacy and carried out in accordance with the Animal Care Guidelines of the National Institutes of Health. All animals were accustomed to the laboratory environment for 3–5 days before entering the study. Experimental study groups were assigned randomly, and the researcher was blind to behavior tests. At the end of the each experiment, the rats were killed using ether anesthesia.

### Epidural catheterization

For epidural administration of drugs, a polyethylene catheter (PE-10: 0.28 mm i.d. and 0.61 mm o.d., Clay Adams, Parsippany, NJ, USA) was implanted into the epidural space under inhaled isoflurane anesthesia (4% for induction and 1% for maintenance) run by an anesthesiameter (Ugo Basile Gas Anesthesia System, Comerio, Italy), as previously described [14]. Briefly, the catheter was gently introduced from the base of the lumbar fifth (L5) spinous process into the lumbar epidural space to a length of about 2 cm. Its tip was thus located at the L3–L4 level. The catheter was then flushed with 100 μl of sterile saline to ensure no leakage into the surrounding tissue. The catheter was tied with a loose knot at a level between the L5 and L6 vertebrae, and was secured to superficial lumbar muscles using 4–0 silk thread. It was then tunneled subcutaneously to the surface of the neck skin and fixed to fascia. Rats displaying obvious hindlimb paralysis were excluded from the study. After completion of the experimental series, we confirmed that the tip of the catheter was most appropriately located at around the L3 level in the epidural space. Furthermore, we confirmed in preliminary experiments, using injection of a dye through the catheter, that the dye was reliably delivered to the epidural (but not the subdural) space around the L3–L4 level in rats. For epidural administration, one hundred microliters of 0.2% ropivacaine mesylate injection, 0.2% ropivacaine sustained release suspension or saline was slowly injected for 30 secs through the epidural catheter, followed by 10 μl of sterile saline.

### Rat model of neuropathic pain

To induce neuropathic pain, the rats were subjected to spinal nerve ligation as described previously [13, 15, 16]. Spinal nerve ligation was used to induce peripheral neuropathy and neuropathic hypersensitivity. The unilateral ligation of two spinal nerves (L5 and L6) was performed under inhaled isoflurane anesthesia (4% for induction and 1% for maintenance) run by an anesthesiameter (Ugo Basile Gas Anesthesia System). The left L5 and L6 spinal nerves were isolated and tightly ligated with 6–0 silk thread. After ligation, the wound was sutured and the rats were allowed to recover. Among the spinal nerve ligated rats, only those with marked unilateral allodynia to mechanical stimulation (hindlimb withdrawal thresholds on the operated side < 8 g) and with no major impairment were included in the study.

### Behavioral assessment of mechanical allodynia and heat hyperalgesia in rats

To evaluate mechanical allodynia [16, 17], rats were acclimated for approximately half an hour to the test environment, a plexiglass box on a metal grid (0.5×0.5 cm). The hindpaw withdrawal threshold (PWT) was measured with a 2450 CE Electronic Von Frey (IITC Life Science Inc, Woodland Hill, CA, USA). The electronic hand-held transducer with a No.15 monofilament (with a measurement range up to 90 g) was applied perpendicularly to the medial surface of the hindpaws, with the force increasing until the rat suddenly withdrew or licked the paw. The lowest force producing a withdrawal response was considered the nociceptive threshold; this was based on three repeated measurements within a 10-min interval and the mean of the three threshold values for each hindpaw, at each time point, was used.

For assessment of heat hyperalgesia, rats were put in a plexiglass box on the elevated glass surface. Following an adaption period of 30 minutes at least, radiate heat was applied to the plantar medial surface of each hindpaw. The hindpaw withdrawal latency (PWL) was measured by a 390G Plantar Test Analgesia Meter (IITC Life Science Inc., CA, USA). To prevent tissue damage, the latency cut-off was set at 30 seconds. The paw withdrawal latency was defined as the time from the onset of radiate heat application to the withdrawal of hindpaws. Both hindpaws were tested independently for three times with a 10-min interval between trials.

### Data analysis and statistical evaluation

The data were expressed as means ± SEM, and there were no missing data. Statistical significance was evaluated by two-way repeated measures analysis of variance (ANOVA) or two-tailed Student t-test in Prism (version 5.01, GraphPad Software Inc., San Diego, CA, USA). The post-hoc Student-Newman-Keuls test was conducted when drug (dose), time and their interaction were observed to be statistically significant. The probability values were two-tailed and the *P*-value meeting the statistical significance criterion was 0.05.

## Results

### Blockade effects of epidural administration of ropivacaine on motor conduction

**Fig 1:**
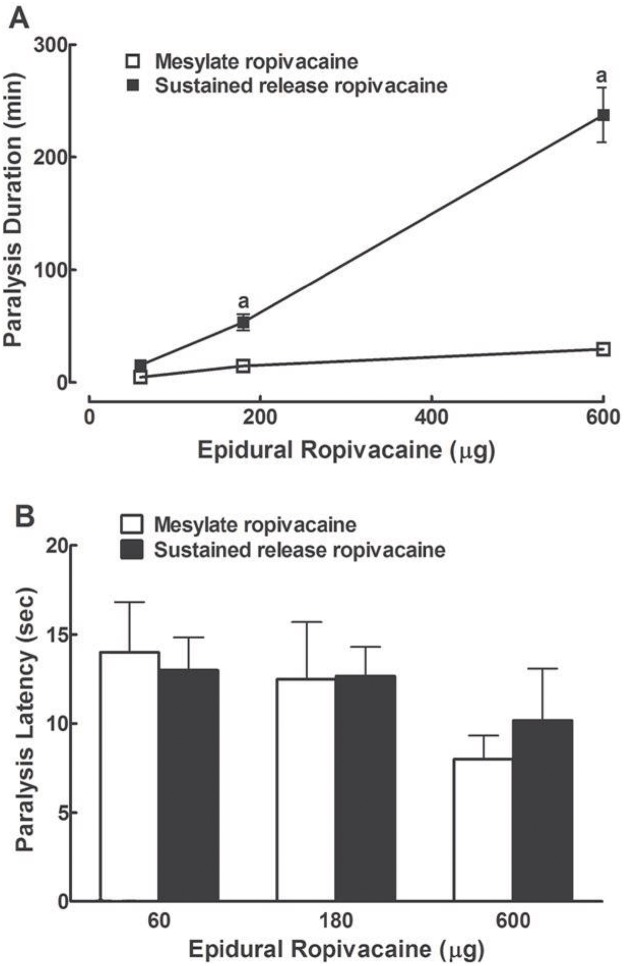
Dose responses of epidural administration of ropivacaine both in mesylate injection and sustained release suspension form on motor paralysis duration (A) and latency (B) in rats. Data are presented as means ± SEM (n = 6 in each group). ^a^ denotes statistically significant difference of ropivacaine sustained release suspension compared to the ropivacaine mesylate group (*P* < 0.05 by a two-way repeated-measures ANOVA followed by a post-hoc Student-Newman-Keuls test).

Motor deficit may affect nociceptive thresholds. To determine the doses and observation period for ropivacaine to produce antinociception, we first examined the motor blockade effect of epidural administration of ropivacaine both in mesylate injection form and sustained release suspension form in eight groups of rats (n = 6 in each group and no animals were excluded from the study). Motor blockade (paralysis) was defined as the inability to negotiate a 60-degree inclined plane [14]. Neither epidural administration of 100 μl saline nor sustained release suspension vehicle produced obvious paralysis in hindlimbs. Epidural injections of mesylate ropivacaine (60, 180 and 600 μg) produced immediate and reversible motor paralysis, which was limited to the hindlimbs in all rats examined as described [14]. The motor blockade effect was dose-dependent, with paralysis duration of 4.6, 14.6 and 29.5 mins, respectively (Fig. 1A). However, the latency for ropivacaine to produce paralysis was dose-independent (i.e., 14, 12.5 and 8 secs, respectively) (Fig. 1B). The motor blockade effect of ropivacaine was consistent with the results in literature [4]. In addition, immediate and reversible motor blockade effects were also observed after epidural injection of ropivacaine sustained release suspension (60, 180 and 600 μg, respectively). The motor blockade effect was also dose-dependent and significantly more prolonged, compared to the mesylate injection, with paralysis duration of 15.4, 53.3 and 237.2 mins, respectively (*P* < 0.05 by a two-way repeated-measures ANOVA, followed by a post-hoc Student-Newman-Keuls test) (Fig. 1A). The paralysis latency was dose-independent (13, 12.7 and 10.2 secs, respectively) (Fig. 1B). We thus selected 200 μg ropivacaine for epidural injection, and measurement of nociceptive thresholds 1.5 hrs post injection and afterwards for further nociceptive studies.

### Blockade effect of epidural administration of ropivacaine on established neuropathic pain

**Fig 2:**
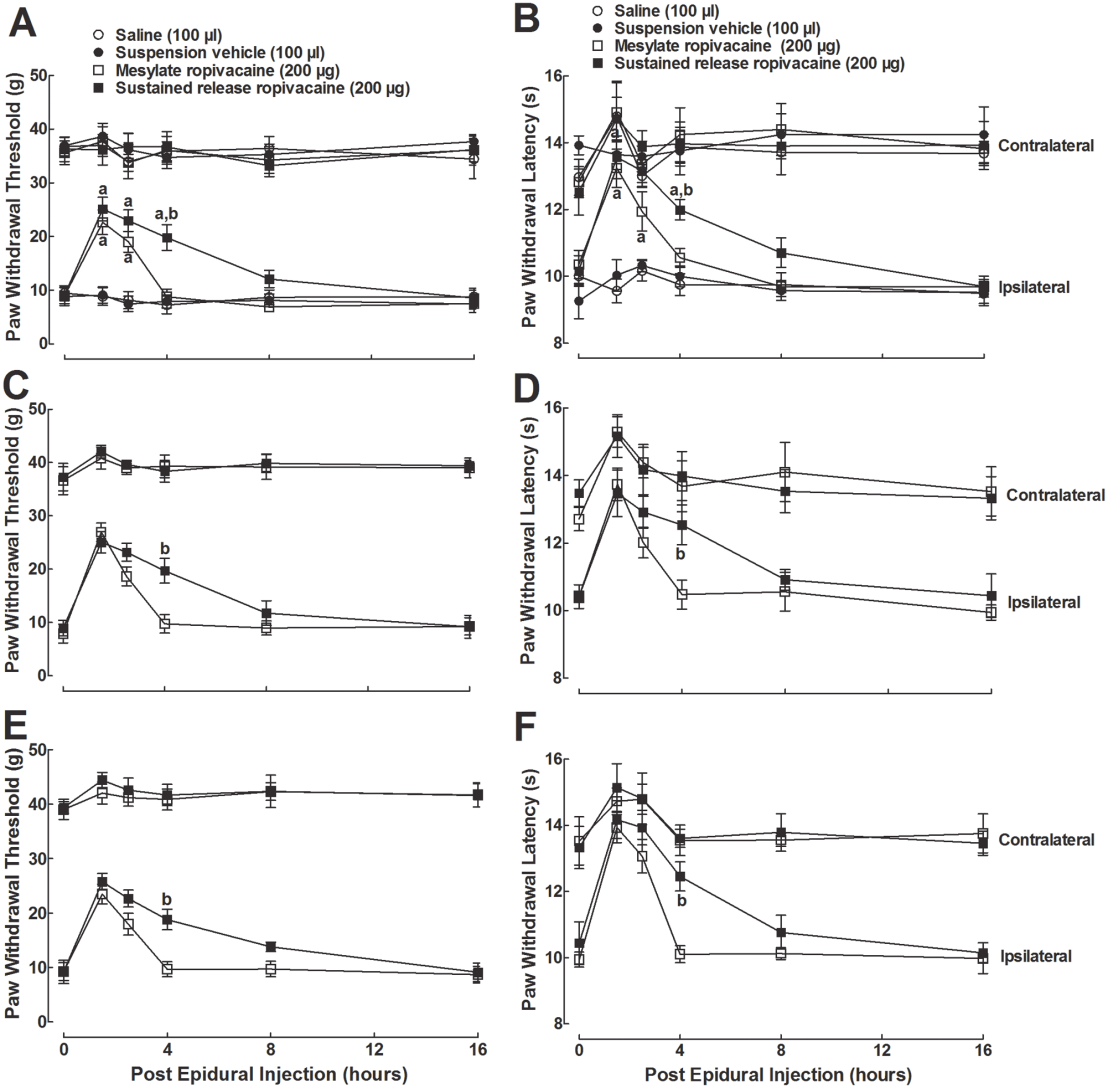
Blockade effects of the subsequent first (A, B), second (C, D) and third (E, F) epidural administration of ropivacaine, both in mesylate injection and sustained release suspension form, on mechanical allodynia and heat hyperalgesia in spinal nerve ligation-induced neuropathic rats. Peripheral neuropathy was induced by unilateral L5/L6 spinal nerve ligation and approximately 7 days later. The rats received repetitive epidural injections for 3 days and the paw withdrawal responses to mechanical and radiate heat stimuli in both the ipsilateral and contralateral hind-limbs were subsequently (with 10-min interval) measured before and 1.5, 2.5, 4, 8, and 16 hrs after epidural injection. Data are presented as means ± S.E.M. (n = 6 in each group). ^a^ and ^b^ denote statistical significance of ropivacaine sustained release suspension compared to the normal saline group and ropivacaine mesylate group, respectively (*P* < 0.05 by a two-way ANOVA followed by a post-hoc Student-Newman-Keuls test).

We examined the blockade effects of epidural administration of ropivacaine both in mesylate injection and sustained release suspension on mechanical allodynia and heat hyperalgesia in neuropathic rats. Approximately 7 days after surgery, four groups of spinal nerve ligated rats (n = 6 in each group) received epidural injection of 100 μl saline, 100 μl sustained release suspension, 200 μg free ropivacaine in both mesylate and sustained release suspension form. The paw withdrawal responses to Von Frey monofilaments and radiate heat were subsequently (with 10-min interval) measured before and 1.5, 2.5, 4, 8, and 16 hrs after epidural injection. As shown in Figs. 2A, 2B, spinal nerve ligation-induced marked mechanical allodynia and heat hyperalgesia when compared with contralateral paws. Epidural treatment with the ropivacaine injection and ropivacaine sustained release suspension did not affect the contralateral paw withdrawal response to mechanical stimulus during the observation period, but they produced a small and short-lived (< 2.5 hrs) inhibition of paw withdrawal latency to radiate heat. On the other hand, ropivacaine in mesylate form significantly blocked mechanical allodynia and thermal hyperalgesia by 52.0% and 70.9%, respectively. The anti-allodynic and anti-hyperalgesic effects were reversible, with a duration of approximately 4 hrs and biological half-lives of 3.0 ± 0.5 hrs and 2.8 ± 0.5 hrs, respectively. Epidural ropivacaine sustained release suspension also produced significant blockade of mechanical allodynia and thermal hyperalgesia by 59.5% and 70.9%, respectively (*P* < 0.05 by a two-way ANOVA followed by a post-hoc Student-Newman-Keuls test). Its blockade magnitude was comparable to that of the mesylate injection form, but its blockade duration (more than 8 hrs) was much longer, with biological half-lives of 5.2 ± 1.4 hrs and 6.4 ± 1.3 hrs for mechanical allodynia and thermal hyperalgesia, respectively (*P* < 0.05 by a two-tailed Student t-test).

The same regimen of ropivacaine was repeated in the same neuropathic rats on the second and third day. The paw withdrawal responses to mechanical and heat stimuli were again subsequently measured 1.5, 2.5, 4, 8, and 16 hrs after epidural injection. For the second treatment, ropivacaine mesylate produced similar mild antinociception (response to heat stimulus) and blockade of mechanical allodynia and heat hyperalgesia, with biological half-lives of 2.7 ± 0.3 hrs and 2.5 ± 0.5 hrs, respectively(*P* < 0.05 by a two-way ANOVA followed by a post-hoc Student-Newman-Keuls test) (Figs. 2C, 2D). Ropivacaine sustained release suspension produced a mild antinociception (response to heat stimulus) but longer anti-allodynic and anti-hyperalgesic effects, with biological half-lives of 6.4 ± 2.5 hrs and 6.8 ± 2.9 hrs, respectively (*P* < 0.05 by a two-tailed Student t-test). During the third treatment, ropivacaine in the mesylate form and sustained release suspension produced similar mild antinociception (heat stimulus) and anti-allodynic and anti-hyperalgesic effects (*P* < 0.05 by a two-way ANOVA followed by a post-hoc Student-Newman-Keuls test), with biological half-lives of 2.7 ± 0.3 and 3.0 ± 0.4 hrs for ropivacaine injection, and 6.0 ± 2.2 and 6.2 ± 1.7 hrs for ropivacaine suspension (*P* < 0.05 by a two-tailed Student t-test) (Figs. 2E, 2F).

### Preemptive effects of the epidural administration of ropivacaine on the development of neuropathic pain

**Fig 3:**
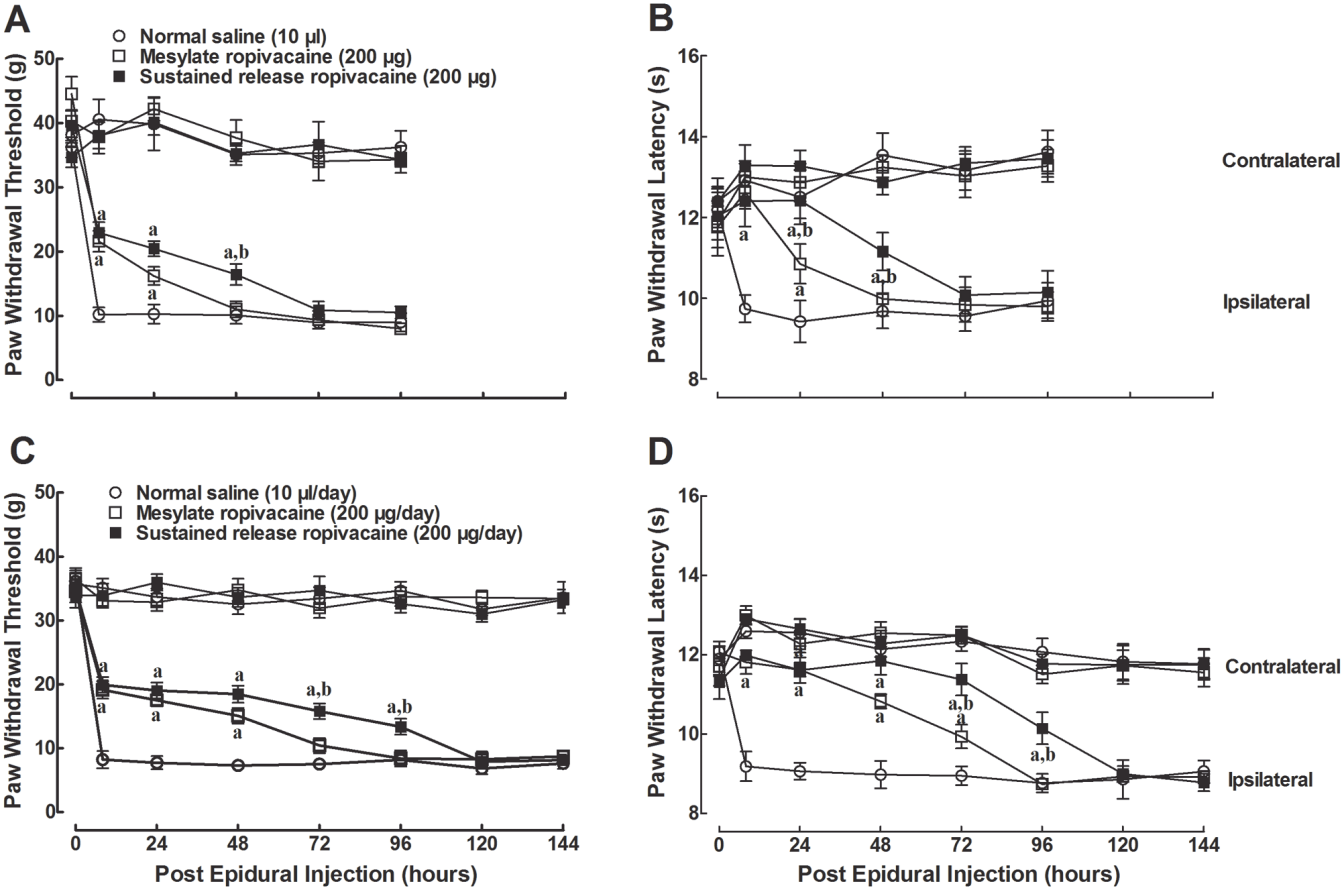
Preemptive effects of epidural administration of ropivacaine both in mesylate injection and sustained release suspension form on mechanical allodynia and heat hyperalgesia in spinal nerve ligation-induced neuropathic rats. The rats received single-dose (**A, B**) or multi-daily (**C, D**) epidural administrations and underwent L5/L6 spinal nerve ligation surgery one hour after the final treatment. The paw withdrawal responses to mechanical and radiate heat stimuli in both the ipsilateral and contralateral hindlimbs were subsequently (with 10-min interval) measured 8 hrs after surgery and afterwards. Data are presented as means ± S.E.M. (n = 6 in each group). ^a^ and ^b^ denote statistical significance of ropivacaine sustained release suspension compared to the normal saline group and ropivacaine mesylate group, respectively (*P* < 0.05 by a two-way ANOVA followed by a post-hoc Student-Newman-Keuls test).

To test the preemptive effects of single epidural administration on the development of mechanical allodynia and heat hyperalgesia in neuropathic rats, three groups of rats (n = 6 in each group) received a single epidural injection of 100 μl saline or 200 μg free ropivacaine in either the mesylate form or sustained release suspension form one hour before spinal nerve ligation. The paw withdrawal responses to Von Frey monofilaments and radiate heat were subsequently (with 10-min interval) measured before surgery, 8 hrs after surgery and afterwards. As shown in Figs. 3A, 3B, mechanical thresholds and withdrawal latency in ipsilateral paws in saline-pretreated rats began to decrease 8 hrs or earlier after the spinal nerve ligation, and were maintained during the observation period of 4 days. However, the epidural administration of ropivacaine mesylate significantly delayed the development of mechanical allodynia and heat hyperalgesia. The responses to mechanical and radiate heat stimuli were approximately half those of saline-treated rats and reached the saline control level 2 days after the surgery, with biological half-lives of 24.2 ± 7.1 and 24.1 ± 6.1 hrs, respectively. A single epidural administration of ropivacaine sustained release suspension produced a more profound delay on the development of mechanical allodynia and heat hyperalgesia (*P* < 0.05 by a two-way ANOVA followed by a post-hoc Student-Newman-Keuls test). Although the responses to mechanical and heat stimuli were approximately half those of saline-treated rats at 8 hrs post-operation, they reached the saline control level 3 days after the surgery, with biological half-lives of 42.2 ± 8.3 and 44.4 ± 4.6 hrs, respectively. They were significantly longer than those of the mesylate form (*P* < 0.05 by a two-tailed Student t-test).

We next observed the preemptive effects of repetitive epidural administration of ropivacaine. Three groups of rats received multiple daily epidural injections of saline (100 μl/day) and free ropivacaine (200 μg/day) in the mesylate form or the sustained release suspension form for 3 days. The rats underwent spinal nerve ligation surgery 1 hr after their last treatment. Paw withdrawal responses to mechanical stimulus and radiate heat were subsequently (with 10-min interval) measured before surgery, 8 hrs after surgery and afterwards. As in Figs. 3C, 3D, the withdrawal responses in ipsilateral paws immediately began to decrease 8 hrs or earlier after spinal nerve ligation in saline-pretreated rats, and were maintained during the observation period of 6 days. The paw withdrawal responses to mechanical stimulus and radiate heat in ropivacaine mesylate multi-daily pretreated rats returned to the saline control level 3 and 4 days after surgery, and had biological half-lives of 48.6 ± 7.9 and 46.3 ± 7.1 hrs, respectively. Multiple daily epidural administration of ropivacaine sustained release suspension prolonged the preemptive effects (*P* < 0.05 by a two-way ANOVA followed by a post-hoc Student-Newman-Keuls test). These paw withdrawal thresholds and withdrawal latencies reached the saline control levels 5 days after surgery, with biological half-lives of 75.3 ± 15.9 and 78.5 ± 7.4 hrs respectively (*P* < 0.05 by a two-tailed Student t-test).

## Discussion

Neuropathic pain is a persistent and intractable pain syndrome caused by trauma or disease affecting the nervous system, such as postherpetic neuropathy or diabetic neuropathy [18, 19]. It is characterized by a combination of spontaneous pain, allodynia, and hyperalgesia [20, 21]. Hyperexcitability after a peripheral nerve injury is considered to be a principal feature of the underlying pathophysiology associated with neuropathic pain, and continuous activity in nociceptors has been shown to be necessary for the maintenance of allodynia and hyperalgesia in humans [22]. Neuropathic pain remains a major issue in clinical practice because of the limited and variable effectiveness of existing analgesics. Cumulative evidence indicates that sodium channels accumulate abnormally within the axons of neuromas [23]. which is responsible for the generation and propagation of action potential in excitable cells [20] and plays a role in the pathogenesis of neuropathic pain [24]. Systemic or topical administration of local anesthetics has attenuated ectopic discharges and suppressed the expression of the neuronal pain marker c-Fos in the spinal cord dorsal horn [25–28]. Intrathecal and epidural administration of a single dose or repeated doses of local anesthetics have been used to alleviate chronic pain in humans [29–31] and experimental animals [32, 33].

Ropivacaine is a long-acting and potent local anesthetic with a sodium channel blockade property nearly 8 times more potent than lidocaine [34, 35]. This study characterized several features of epidural ropivacaine anti-hypersensitivity in neuropathic pain. 1) The anti-allodynic and anti-hyperalgesic effects of ropivacaine mesylate given epidurally was independent of its motor blockade effect, as its anti-hypersensitivity effect still existed after the disappearance of bilateral paralysis. This conclusion is further supported by the fact that epidural ropivacaine did not largely alter the withdrawal responses to mechanical and thermal stimuli measured simultaneously in contralateral paws particularly 3 hr after injection. The results support the notion that local anesthetics produce analgesia and anti-hypersensitivity at doses below those that block motor nerve impulse propagation [36, 37] and that ropivacaine has a good sensory-motor differential blockade property [5]. 2) The single epidural injection of 200 μg ropivacaine blocked spinal nerve ligation-induced hypersensitivity by approximately 50% with a biological half-life of 2.8 hrs. In contrast, it only had a slight and short-lived inhibition on acute nociceptive pain reflected by paw withdrawal responses to thermal but not mechanical stimulus in contralateral paws. The selectivity of ropivacaine on neuropathic pain behaviors is consistent with previous electrophysiological findings that ropivacaine, at doses effectively blocking ectopic discharge and suppressing c-Fos expression in the spinal dorsal horn, failed to block the initiation or propagation of impulses by electrical stimulation and only minimally affected normal sensory receptor activity [38]. 3) Multiple daily epidural injections of ropivacaine did not induce tolerance or potentiation to anti-hypersensitivity, as the magnitude and duration of its anti-hypersensitivity were basically the same for each of the three daily treatments. It has been reported that repetitive epidural ropivacaine over 3 days could significantly reduce thermal hyperalgesia for at least 2 days after the final dose due to potentiation [39]. Although the reason for the discrepancy is not known, our results suggest that multi-daily epidural administrations of ropivacaine do not induce tolerance to anti-hypersensitivity and are appropriate for long-term use.

The more selective contribution from the current study is that sustained release significantly prolongs spinal ropivacaine anti-hypersensitivity in both established and developing neuropathy, which is in good agreement with the physicochemical properties of ropivacaine sustained release suspension [10–12]. A drug is usually released from an appropriate drug delivery system at the injection site and its therapeutic activity is exerted after its entrance into the target site [40]. The oil controlled epidural delivery system provides sustained drug availability in the epidural space for spinal absorption, prolonging its duration of action. This is assumed to be primarily dependent on partition from the lipophilic phase to the tissue fluid followed by passive transport to the capillaries [7, 8]. Thus, a longer duration of ropivacaine activity is obtained when ropivacaine free-base is slowly but gradually released from injectable castor oil. Indeed, pharmacokinetic studies have shown that the elimination half-life of ropivacaine from the sustained release suspension is significantly longer than that of the mesylate injection [11]. Compared to clinically used sequential epidural bolus injection or bolus injection followed by infusion, the oil depot formulation offers a useful alternative to overcome the problem of relatively short duration of analgesics and epidurally administered ropivacaine. The inability of ropivacaine to induce analgesic tolerance was still valid after multiple daily exposures to the sustained release.

The perioperative applications of local anesthetics offer potential benefits, including suppression of noxious stimulation occurring during intraoperation, attenuation of the surgical stress response and stabilization of intraoperative hemodynamics [41, 42]. It is likely that noxious stimulation, initiated intraoperatively, can contribute to central sensitization that occurs postoperatively. Many clinical studies have embraced the concept that epidural anesthesia offers a preemptive analgesic effect [43, 44]. Our study provides further evidence that prior epidural injection of ropivacaine significantly blocks pain development in spinal nerve ligation-induced neuropathic pain in a post-operative pain model. Single and 3-day epidural injection of ropivacaine mesylate before surgery blocked the onset of mechanical allodynia and heat hyperalgesia, bringing the biological half-lives of less than 4 hrs to approximately 1 and 2 days, respectively. These results are consistent with previous findings that administration of low-dose local anesthetic produces preemptive analgesia in the development of postoperative pain in different pain models [43, 45–47]. Moreover, single and 3-day epidural injection of ropivacaine sustained release suspension prior to surgery delayed the biological half-lives further to 2 and 3 days, respectively. The increase in preemptive effects is clinically significant particularly when the species factor is considered, in that humans generally (although not always) metabolize drugs more slowly than rodents by a percentage of 10–30% or more [48].

## Conclusion

To conclude, our results indicate that the epidural administration of ropivacaine produces mild and short-lived antinociception, but blocks neuropathic pain without induction of analgesic tolerance, and significantly delays the development of neuropathic pain. Much more significantly, epidural ropivacaine sustained release suspension blocks established neuropathic pain and delays the onset of neuropathic pain. Our results suggest that the preemptive effect of epidural ropivacaine sustained release formulation would provide clinically significant benefits for patients in reducing postoperative pain and potentially reducing morphine use, although further studies are needed to examine possible neuronal toxic effects of the epidural ropivacaine sustained release formulation of ropivacaine.
